# Salinity-driven niche differentiation within the aquatic Luna-1 subcluster

**DOI:** 10.1093/ismeco/ycaf122

**Published:** 2025-07-16

**Authors:** Annie G West, Jian Sheng Boey, Hwee Sze Tee, Kim M Handley

**Affiliations:** School of Biological Sciences, University of Auckland, Auckland 1010, Auckland, New Zealand; Institute of Ecology and Evolution, School of Biological Sciences, University of Edinburgh, Edinburgh EH9 3FL, United Kingdom; School of Biological Sciences, University of Auckland, Auckland 1010, Auckland, New Zealand; School of Biological Sciences, University of Auckland, Auckland 1010, Auckland, New Zealand; School of Biological Sciences, University of Auckland, Auckland 1010, Auckland, New Zealand

**Keywords:** *Actinomycetota*, estuary, osmoadaptations, photoheterotrophy

## Abstract

Salinity imposes a major barrier to microbial dispersal and colonization due to the requirement for osmoadaptations to maintain cell turgor and protein stability. Estuaries may facilitate infrequent evolutionary transitions between freshwater and marine habitats, which are characterized by differences in both salinity and resource availability. Here we illustrate niche differentiation of the *Actinomycetota* Luna-1 subcluster sister lineages within an estuarine system: freshwater-adapted *Rhodoluna* and saltwater-adapted *Aquiluna*. Comparative genomic and transcriptomic analyses highlighted key differences in osmoregulation, photoheterotrophy, and nutrient acquisition. Both genera are differentiated by mechanisms for osmoregulation, phosphate and iron uptake, and carbohydrate utilization, and by their rhodopsin preference (actinorhodopsin or heliorhodopsin). To clarify which traits are habitat versus lineage specific, we investigated the global distribution of Luna-1 subcluster taxa. The two constituent genera are both more commonly known from freshwater sources, although there are reports of *Aquiluna* isolated from saltwater. Results here confirm that *Rhodoluna* is almost exclusively freshwater-derived. *Aquiluna* instead comprises distinct clades of predominantly freshwater- or saltwater-derived taxa, with approximately half of *Aquiluna* representing slight halophiles from brackish and marine waters. Consistent with observations from the estuary, traits associated with osmoregulation and photoheterotrophy (rhodopsin preference and carbohydrate utilization) differentiated saltwater *Aquiluna* and freshwater members of the global dataset (both *Aquiluna* and *Rhodoluna*), and are therefore likely to be habitat rather than lineage-specific traits. Together, findings demonstrate various genomic characteristics enabling habitat-based niche differentiation between and within lineages of the Luna-1 subcluster, providing insights into microbial adaptation across salinity gradients.

## Introduction

Estuaries offer unique conditions for niche differentiation and interaction among aquatic microorganisms. Freshwater originating from rivers and streams is often rich in organic matter and nutrients from terrestrial sources, including soil erosion, plant material, and anthropogenic runoff [1]. High inputs of nitrogen (e.g. ammonium and nitrate) and allochthonous organic carbon fuels heterotrophic microbial activity in upstream estuarine zones [1]. Marine water is comparatively rich in phosphorus and sulfate (but depleted in nitrogen) [2], and organic carbon is primarily derived from phytoplanktonic primary production [3]. The resulting estuarine ecosystem represents a mixing zone that promotes photosynthesis and nutrient metabolism [2, 4]. Alongside resources, salinity is a major factor influencing estuarine community composition [4–6].

In general, salinity imposes one of the most significant barriers to microbial dispersal and colonization [7–9]. The ability to regulate osmotic pressure and maintain cellular function is energetically costly and requires specialized adaptations [10–13]. Osmoadaptations differentiating brackish and marine taxa from freshwater taxa include those that maintain low cytoplasmic salinity via sodium export and cell turgor via the uptake or synthesis of compatible solutes [4, 6], and stabilize proteins through higher acidity (i.e. higher ratios of acidic versus basic amino acids) [14, 15]. Accordingly, research suggests evolutionary transitions from freshwater to marine habitats within a lineage are rare, although brackish (intermediate salinity) environments likely serve as a conduit for such events [9, 15]. Microbial communities inhabiting freshwater and saltwater environments are taxonomically and functionally distinct, yet closely related taxa often exist in both environments [9, 14–17]. SAR11/*Pelagibacterales*, the most abundant marine microorganism on earth, is arguably the most well-known example of this phenomenon, where a number of distinct subclades are also adapted to freshwater and brackish environments [18–20].

*Actinomycetota* represent another potential source of brackish-adapted lineages, albeit originating from freshwater [21]. *Actinomycetota* are often the most dominant members of freshwater habitats [22, 23]. They are also one of the main phyla observed in brackish waters, alongside *Pseudomonadota* (notably *Pelagibacterales*) and *Bacteroidota* [4, 15, 20, 24, 25]. Among *Actinomycetota* found in brackish water, and that potentially derive from freshwater, are members of the *Microbacteriaceae* Luna-1 subcluster (one of two subclusters of the broader Luna cluster [26]), and the actinomycetes acI lineage (Ca. Nanopelagicales). Both Luna-1 [26–29] and acI [30, 31] are widespread in freshwater environments, and are most commonly considered freshwater lineages. The presence of brackish-adapted members of both lineages may therefore represent examples of transitions from freshwater to saltwater [21, 24, 32, 33]. Despite evidence for the diversification of typically freshwater lineages into neighboring saline environments, there is limited study of the underlying mechanisms facilitating such adaptation.

Here, we determined the distribution and genetic traits of the *Actinomycetota* Luna-1 subcluster in relation to salinity. The Luna-1 subcluster comprises two closely-related sister genera, *Aquiluna* and *Rhodoluna*, which were first isolated from freshwater lakes and ponds [26, 27, 34]. Both genera are characterized as photoheterotrophs, with small genomes (<2 Mbp) and cell sizes (<0.22 μM) [27–29]. Although typically considered freshwater taxa, *Aquiluna* has been identified in saltwater [32]. Our study illustrates the niche differentiation of freshwater-adapted *Rhodoluna* and saltwater-adapted *Aquiluna* species within an estuary, wherein, for the purposes of this study, “saltwater” refers to brackish and marine salinities (0.5–35 ppt). Results highlight differences in their genetic traits and transcriptional activity associated with photoheterotrophy, nutrient acquisition, and osmoregulation. Furthermore, using publicly available Luna-1 subcluster genomes, we compared the phylogenetic distribution of saltwater- versus freshwater-adapted members of the Luna-1 subcluster and identified trait differences based on either habitat salinity or lineage.

## Materials and methods

### Dataset generation

**Figure 1:**
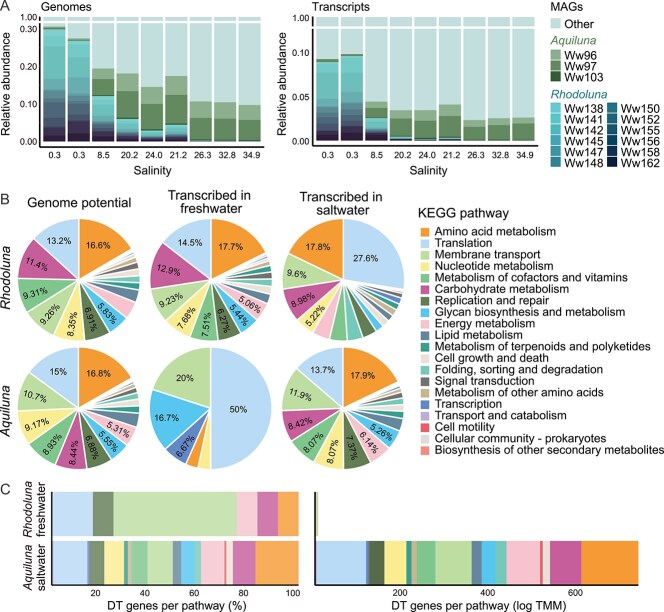
Metabolic capacity and transcriptional activity of Luna-1 subcluster members, and their spatial distribution within the Waiwera estuary. (A) Relative MAG and transcript abundance across the estuary. *Aquiluna* MAG Ww96 is included here for comparison, but is excluded from all other figures and analyses due to its high level of genome incompleteness (10.7%). (B) Proportions of genes encoded per genus associated with KEGG metabolic pathways (“genome potential”), and genes transcribed in freshwater (sites 1 and 2) and saltwater (sites 3 to 9). (C) Genes differentially transcribed (DT) per KEGG pathway in freshwater versus saltwater. DT genes shown are those significantly more highly transcribed in freshwater for *Rhodoluna* and in saltwater for *Aquiluna*.

Nine water samples (>0.22 μm cell fraction) were collected from the Waiwera estuary (Auckland, New Zealand)—a shallow tidal lagoon estuary—as described previously [4]. In brief, each sample was collected from one of nine sites along a 5 km transect (1–2 freshwater [salinity 0.3 ppt], 3–7 brackish [salinity 8.5–26.3 ppt], 8–9 marine [salinity 32.8–34.9 ppt]; Fig. 1A), and from 0.2 to 1 m below the air–water interface. Metagenomic and metatranscriptomic data were generated, and metagenomes assembled, as also described previously [4]. Metagenome-assembled genomes (MAGs) from the nine sites (*n* = 1084, Supplementary Information) were assigned taxonomy with GTDB-Tk v2.4.0 (GTDB 214 release) [35] and filtered for contamination (<5% after accounting for strain heterogeneity) and completeness (>50%), yielding 29 Luna-1 subcluster MAGs for further analysis from an initial 37 (Supplementary Table S1). CheckM v1.2.3 [36] was used to estimate strain heterogeneity and contamination (Supplementary Table S1). CheckM2 v1.0.1 [37] estimates were generated for comparison.

### Relative abundance and transcription

To determine MAG relative abundances, the filtered dataset was dereplicated at 98% average nucleotide identity (ANI) using dRep v3.4.2 [38]. Metagenomic reads were mapped via BBMap v39.01 [39] against the non-redundant set of 646 MAGs (ambiguous = best, minid = 0.98). MAG coverages per sample were determined as genome length/mapped read length, and were used to calculate MAG relative abundance per sample (i.e. percent abundance based on MAG coverage). To determine transcription associated with MAGs, trimmed metatranscriptome reads were mapped using the same approach and assigned to predicted genes using featureCounts 2.0.6 [40] (≥5 reads mapped required to be considered transcribed). Reads were trimmed using Trimmomatic v0.39 [41] with a 4 bp sliding window, 30 Phred score threshold, and ILLUMINACLIP for adapter removal, retaining reads 50–115 bp long. Differentially transcribed genes were identified with edgeR (Bioconductor v3.15, R v4.2.1) [42]. Read counts were also normalized using the trimmed mean of M values (TMM) method [43] for visualization of the transcription of genes associated with particular metabolic traits.

### Strain diversity and phylogeny of Luna-1 subcluster genomes

The microdiversity of Luna-1 subcluster MAGs was explored using inStrain v1.5.7 [44]. Reads were mapped to dereplicated Luna-1 subcluster MAGs with Bowtie2 v2.4.5 (−sensitive mode) [45] and inStrain was run on all nine samples with profile and compare functions. To determine the phylogenetic placement of the Luna-1 subcluster MAGs, an alignment was made of core single-copy proteins from the 29 nondereplicated MAGs, 101 publicly available Luna-1 subcluster genomes (each species representative in the GTDB 09-RS220 release, Supplementary Table S1), and a multi-phyla outgroup (Supplementary Information) using GTDB-Tk v2.4.0 [35]. A tree was constructed using IQ-TREE v.2.2.2.2 [46] with 1000 ultrafast bootstrap replicates and ModelFinder for best-fit model selection, and visualized in iTOL v6.9.1 [47]. Pairwise ANI among genomes within each genus was computed using pyani v0.2.12 with BLAST alignments [48].

### Annotation and pangenome analysis

Gene and protein sequences were predicted from the combined Luna-1 subcluster dataset (29 MAGs and 101 reference genomes) and annotated via DRAM v1.4.6 [49] with KEGG [50] and Pfam [51] databases. Encoded amino acids proportions were determined from predicted protein sequences [52]. Secreted proteins were identified with signalP v6.0 [53]. Pangenomes of *Rhodoluna* and *Aquiluna* genera were determined with the anvi’o v8 pangenome snakemake pipeline (MCL inflation −5) [54]. Functional enrichment analysis among *Aquiluna* genomes was implemented as per Shaiber *et al.* [55] in anvi’o.

### Rhodopsin phylogeny and gene synteny

Rhodopsin protein sequences identified in Luna-1 subcluster genomes were aligned against known reference sequences for heliorhodopsin, actinorhodopsin, xanthorhodopsin, *Exiguobacterium sibiricum* rhodopsin (ESR), archaeal rhodopsin, and fungal rhodopsin using MAFFT v.7.505 [56]. Four novel rhodopsin genes from *Microcella* and *Pontimonas* [57] were also included as both genera are members of the same family as Luna-1 (*Microbacteriaceae*), although their rhodopsins did not cluster with other *Actinomycetota* bacteriorhodopsin-like sequences. A tree was built using IQTREE v.2.2.2.2 (settings as above), subsetted with ape v5.7.1 [58] to highlight diversification of MAG-derived rhodopsins, and visualized via iTOL. Pairwise phylogenetic distances among Luna-1 subcluster actinorhodopsin and heliorhodopsin genes were calculated using the tree branch lengths with ape’s cophenetic.phylo function.

Gene synteny plots were used to visualize homology among genes proximal to carotene synthesis. Selected genomes were subsetted to the 20 preceding and subsequent genes surrounding those of interest, and the resulting amino acid files aligned with BLAST v2.13.0 [59]. Contigs were plotted with the gggenomes package v1.0.1 [60].

### Graphical and statistical analyses

Additional statistical and graphical analyses were undertaken in R v.4.3.2 [61]. Heatmaps were generated using the gplots heatmap.2 package v3.1.3.1 [62] and pheatmap v1.0.12 package [63] as specified below. Pie and bar plots were generated using ggplot2 v.3.5.1 [64]. Spearman’s correlations were calculated with the cor.test function in *R*, and Welch two-sample *t*-tests with the wilcox.test function.

## Results and discussion

### Spatial differentiation of the Luna-1 subcluster within an estuary

We recovered 29 MAGs from across the Waiwera estuary that were taxonomically assigned to the *Microbacteriaceae* Luna-1 subcluster (19 *Rhodoluna* and 10 *Aquiluna*; 83% average completeness, range 55%–98%, <5% contaminated; Supplementary Table S1). Estimated genome sizes (1.2–2.3 Mbp, average 1.6 Mbp, 54% GC) are comparable to Luna-1 species *Rhodoluna lacicola*, *Rhodoluna planktonica*, *Rhodoluna limnophila*, and *Aquiluna borgnonia* (all 1.4 Mbp, average 53% GC) [27–29, 65], and the combined dataset of 130 Luna-1 subcluster genomes (average 1.5 Mbp, 53% GC). The small genome sizes recovered are also consistent with small cell sizes (<0.5 μm long, <1.2 μm wide) of *Aquiluna* species *Ca.* Aquiluna rubra and *A. borgnonia* [27, 29]. All estuarine MAGs belonged to unclassified (novel) species. *Rhodoluna* MAGs comprised greater species-level diversity (ANI threshold of 96.5% [66]) with 12 species identified (~1.5 MAGs/species, 75.0% average ANI overall) versus two *Aquiluna* species (~5 MAGs/species, 85.7% average ANI overall) (Supplementary Figs S1–S2, Supplementary Table S2). However, *Aquiluna* MAGs exhibited greater diversity per species and higher strain heterogeneity (five *Aquiluna* versus one *Rhodoluna* MAG with >10% heterogeneity; Supplementary Table S1). *Aquiluna* MAGs also hosted more SNVs (on average 33 515 or 40 393 normalized to MAG relative abundance) than *Rhodoluna* MAGs (8580 or 12 338 normalized; Supplementary Fig. S3, Supplementary Table S3). Thus, while fewer *Aquiluna* MAGs and proportionally fewer species were identified from the estuary, *Aquiluna* populations were more heterogeneous.

*Rhodoluna* and *Aquiluna* overlapped spatially in the estuary (based on MAG community abundances), but were each most abundant in distinct salinity zones (Fig. 1A, Supplementary Table S4). *Rhodoluna* populations dominated freshwater samples, comprising up to 28% of microbial community abundance (*Rhodoluna* Ww142 was most abundant; up to 5.8%). *Aquiluna* comprised up to 10% of saltwater communities (brackish and marine), with *Aquiluna* Ww97 the most abundant member of the genus across all seven saline sites (up to 5.7% community abundance). SNV diversity and overall gene transcription were likewise highest in freshwater for *Rhodoluna* and saltwater for *Aquiluna* (Fig. 1A, Supplementary Fig. S3). Most genes were exclusively transcribed by *Rhodoluna* in freshwater (8131/10 817 genes) and *Aquiluna* in saltwater (1252/1321 genes; Supplementary Table S5). Overall, results showed clear spatial niche differentiation between Luna-1 subcluster genera, corresponding to large differences in salinity and nutrient availability [4].

Despite the low relative abundances of *Rhodoluna* at saline sites and *Aquiluna* at freshwater sites, both genera were transcriptionally active in their “non-preferred” habitats (Fig. 1A and B), indicating some degree of saltwater or freshwater tolerance by *Rhodoluna* and *Aquiluna*, respectively. However, the functional composition of genes transcribed was strongly differentiated between non-preferred and preferred habitats, particularly for *Aquiluna* (Fig. 1B and C). The composition of metabolic functions (KEGG assignments) based on the number of genes present and transcribed was more highly correlated in preferred habitats (Spearman’s correlations = 0.98 for *Rhodoluna*, 0.99 for *Aquiluna*; *P*-values <0.001) versus non-preferred habitats (0.90 for *Rhodoluna*, 0.06 for *Aquiluna*; *P*-values <0.001 and 0.9, respectively) (Supplementary Fig. S4). In preferred habitats, amino acid metabolism, translation, membrane transport, nucleotide metabolism, metabolism cofactors and vitamins, and carbohydrate metabolism were the most abundant functional categories based on genes present and transcribed (Fig. 1B). It is unclear whether the streamlined genomes of these genera could account for the level of congruence between gene transcription and presence in preferred habitats. Streamlined free-living or symbiotic/parasitic prokaryotes with small genome sizes tend to have fewer non-core genes and fewer genes for transcriptional regulation, such as repressors and RNA polymerase binding sigma factors [67–69]. Thus, there is a greater tendency for genes to be constitutively expressed in these organisms, although transcription would nonetheless be moderated by factors such as growth rate, concentrations of free RNA polymerase per cell, and promoter strength [70–72]. However, whether such factors could account for the difference in gene transcript composition observed between freshwater and saltwater habitats in this study remains unknown. The extent to which regulatory mechanisms could have been lost by members of the Luna-1 subcluster also warrants future investigation.

**Figure 2:**
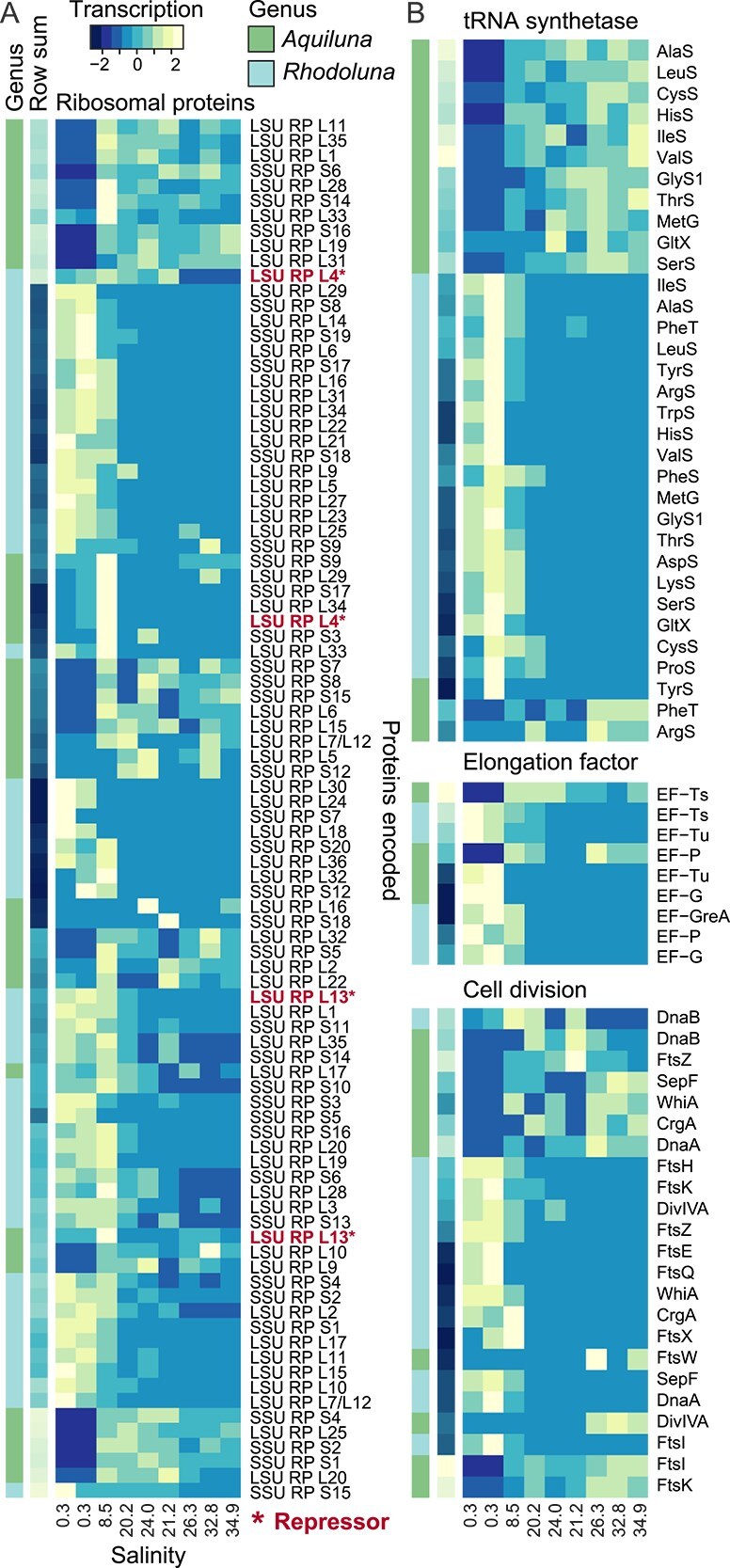
Transcription of genes by Luna-1 taxa across the estuary that are associated with protein synthesis and growth. (A) Ribosomal proteins. (B) tRNA synthetase, elongation factors, and cell division. Ribosomal proteins that function as transcriptional repressors (L4 and L13), with notably high gene transcription in non-preferred habitats, are shown in bold red font. Transcript values (TMM normalized counts) were summed by genus per gene per sampling site (see Supplementary Table S5 for individual gene transcription TMM values and raw counts), and plotted using heatmap.2. Transcript values were scaled by row with mean equal to zero and a standard deviation of one. Rows were ordered based on row means using the Rowv function.

Based on transcriptome analyses, results suggest a highly restricted metabolism for *Rhodoluna* in saltwater, and *Aquiluna* in freshwater, with little investment in growth. Gene transcription was disproportionately associated with ribosomal proteins (Fig. 1B). This was most evident for *Aquiluna*, where translation (notably ribosomal proteins) accounted for 50% of transcription compared to just 13.7% in saltwater. *Aquiluna* genes transcribed in freshwater were also disproportionately associated with membrane transport (20%, mostly genes associated with Sec translocase and amino acid transport *livGFK, yidC*, and *secY*) and glycan metabolism (16.7%, genes *uppS* and *ddl*) (Supplementary Table S5). Although ribosomal protein-related transcription comprised a large fraction of Luna-1 subcluster transcription in non-preferred habitats, it comprised a small portion of community gene transcription (e.g. *Aquiluna* averages: 5.7 TMM freshwater, 170.1 TMM saltwater), and the number of genes transcribed was small (Fig. 2A). Some ribosomal protein-encoding genes transcribed by *Aquiluna* and *Rhodoluna* in non-preferred habitats (L4 and L13, bold red font Fig. 2A) function as autogenous transcriptional repressors that inhibit further ribosomal protein synthesis [73, 74]. Accordingly, genes encoding tRNA ligases (aminoacyl-tRNA synthetases) were almost exclusively transcribed (and many significantly differentially) in preferred habitats where transcription associated with cell division (*ftsIKWZ*, *whiA*, and *crgA*), chromosomal replication (*dnaAB*), and elongation factors (Ts and P for *Aquiluna*; Ts, P, Tu, G, and GreA for *Rhodoluna*) was also appreciably higher (Fig. 2B).

Ribosome numbers and protein translation typically decrease together with cell growth rates [75], while a sizable and largely inactive ribosome pool is maintained in slow-growing cells for stress management and/or to poise cells for a return to optimal (e.g. resource-rich) conditions [75, 76]. The predominance of ribosomal protein transcription by *Aquiluna* in freshwater, alongside a small transcriptional investment in elongation factors (0.4 TMM on average for EF-Tu and EF-G in freshwater versus 28.9 TMM for EF-Ts and EF-P in saltwater; Fig. 2B), potentially reflects cell maintenance under non-optimal conditions and preparation for a rapid metabolic response to favorable future conditions. Elongation factors can also play a role in osmoregulation. The EF-Tu product increases in *Arabidopsis* cells experiencing hyperosmotic stress [77], and is released in a burst-like fashion from bacterial cells under low osmolarity (downshock) to alleviate internal pressure and membrane tension [78]. A transcriptional investment in translation machinery and elongation factors could thus be helpful in an estuary, subject to periodic tidal fluctuations.

### Multiple *Aquiluna* species are saltwater-adapted globally

**Figure 3:**
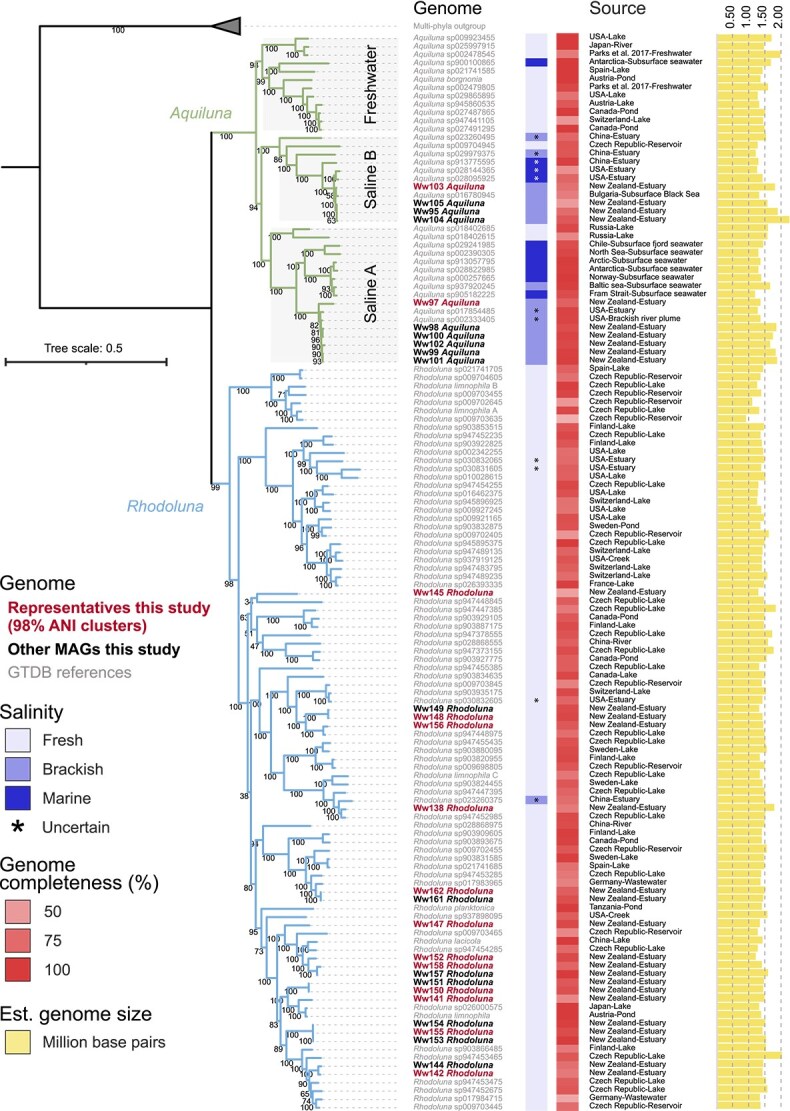
Luna-1 subcluster clades and phylogenetic placement of *Rhodoluna* and *Aquiluna* MAGs within these. The tree was constructed using GTDB alignments of conserved single-copy protein sequences (best-fit model LG + I + R7). *Aquiluna* clades are designated “freshwater,” “saline A,” and “saline B” based on the predominant habitat association of constituent members. Blue squares indicate the respective salinity from which genomes were sampled (asterisks denote putative habitat assignment). Red squares indicate genome completeness estimates derived from CheckM analysis of single-copy marker genes. Yellow bars represent estimated genome sizes.

To place Waiwera-derived Luna-1 subcluster MAGs into a global context, we constructed a phylogenetic tree with our MAGs and all 101 representative genomes for *Rhodoluna* and *Aquiluna* in GTDB. Genomes were assigned a habitat salinity (freshwater, brackish, or marine) based on: (i) the habitat where estuarine MAGs (this study) were most abundant and/or transcriptionally active, or (ii) the “source environment” reported in GTDB metadata for cultured isolates or MAGs/SAGs from a distinct freshwater or saltwater source (e.g. lake or ocean as opposed to estuary) (Supplementary Table S1). Due to the likelihood of transport between salinity zones within estuaries, estuarine MAGs/SAGs with only assembly location available were assigned a putative habitat salinity. The resulting phylogenetic tree and associated habitat salinities (Fig. 3) show that *Rhodoluna* and *Aquiluna* comprise distinct clades, and that these genera differ in their habitat distributions.

Based on currently available isolates and environmental genomes, members of the *Rhodoluna* genus are (almost) exclusively adapted to a freshwater lifestyle. Almost all (*n* = 89/90) representatives to-date were sampled from freshwater with just one putatively brackish population assembled from an estuary in southern China. In fact, 94.4% of all reference *Rhodoluna* genomes were sampled or isolated exclusively from freshwater bodies, such as lakes and rivers. *Rhodoluna* MAGs from the Waiwera estuary were phylogenetically diverse. All were located within, and distributed across, the largest *Rhodoluna* sub-clade along with *R. limnophila*, *R. limnophila*_C, *R. lacicola*, and *R. planktonica*.

In contrast, roughly half of *Aquiluna* genomes were derived from saline environments (*n* = 26/40, with eight putative assignments; Fig. 3). Based on representative genomes, *Aquiluna* clustered into two separate clades, each with either predominantly freshwater- or saltwater-adapted members. The predominantly saltwater clade comprised entirely uncharacterized species distributed across two sub-clades (saline A and B), and includes all estuarine *Aquiluna* MAGs from this study alongside genomes recovered from other brackish and marine environments, such as the Black and Baltic seas and Antarctic seawater. The same three clusters (freshwater, saline A, and saline B) were also evident when comparing pairwise ANIs (Supplementary Fig. S2) and pangenome gene clusters (Supplementary Fig. S5). Results indicate early evolutionary divergence of freshwater and saltwater *Aquiluna* lineages, akin to the deep evolutionary divergence of saltwater and freshwater lineages of *Pelagibacterales* [18, 19], and subsequent diversification within each habitat salinity type.

### Habitat-based differentiation of traits within the Luna-1 subcluster

#### Osmoadaptive traits differentiate freshwater and saltwater Luna-1 taxa

**Figure 4:**
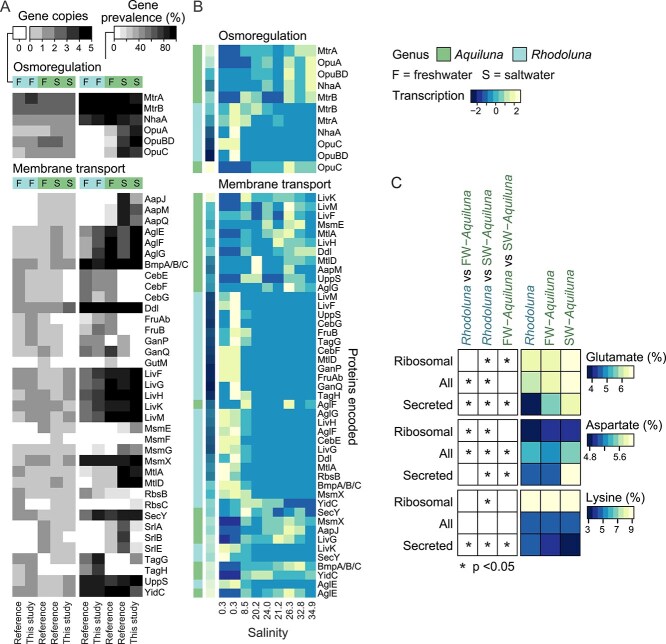
Transcription, copy numbers, and prevalence of osmoregulation and membrane transport genes in Luna-1 subcluster taxa. (A) Pairs of plots showing mean gene copy numbers per genome (left) and gene prevalence per taxa group (right) for *Rhodoluna* and freshwater (F) and saltwater (S) *Aquiluna* (reference genomes and those from this study). Gene copy numbers per genome were adjusted based on genome contamination and completeness prior to calculating averages per taxa group, and are shown unscaled. Rows are ordered alphabetically based on gene name. (B) Gene transcription by Luna-1 taxa in the Waiwera estuary. Transcript values (TMM-normalized counts) were summed by genus per gene per sampling site (see Supplementary Table S5 for individual gene transcription TMM values and raw counts). Transcript values were scaled by row with mean equal to zero and a standard deviation of one, and rows were ordered based on row means. (C) Right plots: Average charged amino acid percentages encoded by Luna-1 genomes for all proteins, ribosomal proteins, and secreted proteins. Data is unclustered hierarchically and unscaled. Left plots: Asterisks indicate significant differences in amino acids encoded between Luna-1 subcluster groups (pairwise Wilcoxon rank sum tests with Holm-Bonferroni correction; *P*-values <0.05). Plots (A–C) were created using heatmap.2.

Saltwater *Aquiluna* genomes (130 genomes from the Waiwera estuary and elsewhere) hosted several genes associated with osmoregulation that were largely or completely absent from *Rhodoluna* and freshwater *Aquiluna* (Fig. 4A, Supplementary Tables S6–S7). Notably, >77% of saltwater *Aquiluna* had genes for the osmoprotectant transport system (*opuABDC*) compared to substantially fewer freshwater *Aquiluna* (<29%) and *Rhodoluna* (6%). The osmoprotectant transport system imports the compatible solute glycine betaine with high affinity [79, 80]. Genes encoding the AapJMQ and MsmEG transporter systems were also more common in saltwater *Aquiluna* genomes, with *aapJMQ* completely absent in *Rhodoluna* (Fig. 4A, Supplementary Information). AapJMQ is a general L-amino acid transport system and high-affinity transporter for L-glutamate [81], which is a compatible solute at low (e.g. brackish) salinities [82]. MsmEG transports sugars, such as raffinose (produced by some plants and algae), which can also serve as a compatible solute [83, 84]. Additionally, saltwater *Aquiluna* genomes more frequently hosted genes encoding uptake and synthesis of the sugar alcohol and compatible solute mannitol (*mtlAD*). Mannitol PTS permease (MtlA) is responsible for the uptake and phosphorylation of mannitol (yielding mannitol-1-phosphate) [85], whereas mannitol-1-phosphate 5-dehydrogenase (*mtlD*) reversibly synthesizes mannitol from the glycolytic pathway intermediate D-fructose-6P [86]. Such variation in compatible solute uptake (or synthesis) mechanisms (Opu, Msm, Aap, and Mtl) points to a divergence in the osmoregulation and substrate utilisation capabilities of Luna-1 subcluster taxa based on habitat salinity.

Accordingly, transcription of various osmoregulation genes by *Aquiluna* was substantially higher in saltwater versus freshwater, and higher than *Rhodoluna* in any part of the Waiwera estuary (Fig. 4B). This included higher transcription of genes encoding the OpuABDC osmoprotectant transport system, AapJM general L-amino acid transport, mannitol (MltA) and raffinose (MsmE) transporters, the NhaA sodium-hydrogen antiporter (Na^+^ export [87]), and actinomycetota MtrAB osmostress response regulator. Of these, *opuAC*, *aapJ*, *mltAD*, and *mtrA* were significantly more highly transcribed by *Aquiluna* MAGs Ww97 and Ww103 in saltwater (Supplementary Table S5). In the MtrA–MtrB two-component system, osmosensing via the MtrB histidine kinase is activated in the presence of cytoplasmic organic solutes [88], and MtrA is reported to regulate essential functions in *Mycobacterium* (*Actinomycetota*), including cell division [89, 90]. The presence of these osmoregulatory genes and transcription in saltwater is consistent with use of the salt-out approach by *Aquiluna* to maintain osmotic stability [12].

A further feature of adaptation to saline conditions is higher protein acidity, achieved through the enrichment of acidic amino acids glutamate and aspartate, and depletion of the basic amino acid lysine [11]. Higher protein acidity is thought to maintain protein stability and function in saline habitats [91]. Accordingly, the genomes of saltwater *Aquiluna* (both our estuarine MAGs and reference genomes) encoded a significantly greater proportion of acidic amino acid aspartate, overall, compared to *Rhodoluna* or freshwater *Aquiluna* (Wilcoxon sum rank test, *P*-values <5.0 × 10^−6^, Fig. 4C; Supplementary Tables S8–S9). This difference was even more pronounced in the secreted protein fraction, where, in addition to aspartate, encoding for glutamate was clearly significantly enriched and lysine significantly depleted in saltwater *Aquiluna* (*P*-values <0.002). In salt-out strategists, proteins with greater proportions of acidic amino acids tend to be those exposed to the external environment [14]. Results therefore indicate that *Aquiluna* populations inhabiting saltwater encode for more acidic secreted proteins as part of the salt-out osmoadaptive strategy. Below we identify non-osmoadaptative traits related to photoheterotrophy that further differentiate Luna-1 subcluster genomes by freshwater or saltwater habitat.

### Habitat-specific strategies for organic carbon acquisition and utilization

**Figure 5:**
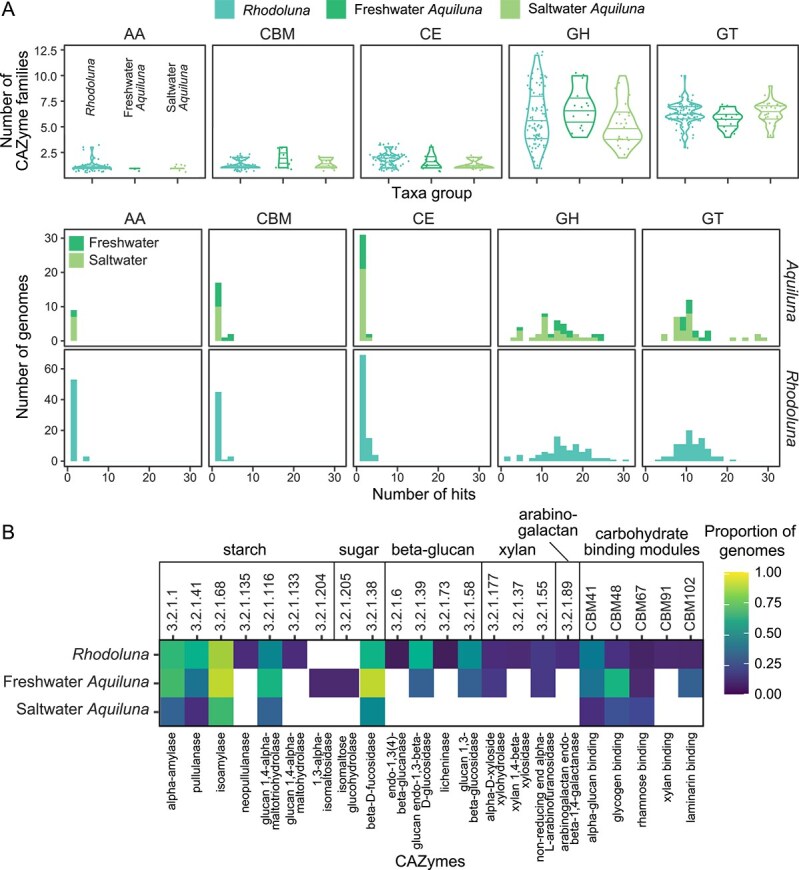
Genetic capacity of Luna-1 subcluster taxa to encode for CAZymes (including genomes from this study and reference genomes). (A) Number of CAZyme families found per genome per taxa group (*Rhodoluna*, freshwater *Aquiluna*, and saline-water *Aquiluna*) (top row). Horizontal lines within the violin plots show the 25%, 50%, and 75% percentiles. The lower two rows of plots show the number of genomes predicted to encode each CAZyme class. No polysaccharide lyases were found in any genome. Abbreviations: Auxiliary activities (AA), carbohydrate-binding modules (CBM), carbohydrate esterases (CE), glycosyl hydrolases (GH), and glycosyltransferases (GT). (B) Distribution of GH and CBM-encoding genes observed across genomes. White cells indicate absence of the gene. The heat map was generated using ggplot with no scaling applied.

Cultures of Luna-1 subcluster taxa assimilate a variety of sugars (alpha-D glucose, D-mannose, sucrose, fructose, turanose, d-galactose, cellobiose, maltose, trehalose, melibiose, gentiobiose, and raffinose) in addition to mannitol, arabitol, inosine, and glucuronamide [28, 29, 65], and are likely to be efficient in scavenging diverse organic carbon resources in natural aquatic systems. Here, we assessed the distribution of carbohydrate degradation and carbon uptake mechanisms across the set of 130 genomes to determine common traits and those differentiating freshwater and saltwater Luna-1 taxa. Carbohydrate utilization capacities were broadly similar between genera and habitat salinities (Fig. 5A, Supplementary Table S10). Analysis of CAZyme enzyme commission (EC) numbers indicated that members of the Luna-1 subcluster universally lack genes for polysaccharide lyases necessary for the degradation of structurally diverse and complex marine polysaccharides, such as alginate [92] and ulvan [93], that may be expected in an estuarine environment. However, both genera have the capacity to degrade alpha-glucans via multiple prevalent GH13 CAZyme-encoding genes, aided by carbohydrate binding modules (CBM; specifically 41 and 48) that bind alpha-glucans such as starch and glycogen (Fig. 5B). These genes were also transcribed by Luna-1 taxa throughout the estuary (Supplementary Table S5). Likewise, some genes for carbon uptake were among those most highly transcribed by both freshwater and saltwater taxa groups in their preferred habitats (Fig. 4B). Notably, *aglE* for alpha-glucoside transport (i.e. import of maltose, trehalose, maltotriose, melezitose, sucrose, and α-methylglucoside) [94] and *livK* for high-affinity branched-chain amino acid transport [95] were highly transcribed by both taxa groups, suggesting prioritization for some of the same substrates despite differences in gene repertoires and habitat.

Despite these commonalities, overall expression of membrane transport-associated proteins within the Waiwera estuary was substantially higher for *Aquiluna* than *Rhodoluna* (mean 2136 TMM for *Aquiluna* in saltwater versus 1523 TMM for *Rhodoluna* in freshwater, or 4.9 times higher after normalizing to genome relative abundance averaged across preferred habitats) (Fig. 4B). In addition, freshwater and saltwater members of the Luna-1 subcluster differ in several mechanisms they have for organic carbon acquisition and utilization. For example, a greater number of saltwater *Aquiluna* host genes involved in uptake of the sugar alcohol glucitol (*srlABE* with/without the *gutM* regulator gene; 52% of saltwater *Aquiluna* versus 21% of freshwater *Aquiluna* and no *Rhodoluna*) (Fig. 4A). Additionally, more saltwater *Aquiluna* genomes host genes for the uptake of mannitol (*mtlA*, 93% of saltwater *Aquiluna* versus 29% of freshwater *Aquiluna* and 18% of *Rhodoluna*) and raffinose (*msmE*, raffinose/stachyose/melibiose transport, 52% of saltwater *Aquiluna* versus 7% of freshwater *Aquiluna* and 4% of *Rhodoluna*), which as discussed above could help cells maintain osmotic balance.

In contrast, freshwater taxa harbored more mechanisms for degrading algal and plant biomass, including genes for degrading beta-1,3-glucans such as laminarin (58% of *Rhodoluna* and 29% of freshwater *Aquiluna* versus no saltwater *Aquiluna*; EC 3.2.1.39, Fig. 5B, Supplementary Table S10). Beta-1,3-glucans often function as storage molecules in microalgae and diatoms that are highly abundant in estuaries [4] and marine environments [96], and are easily degraded to glucose [96, 97]. Some *Rhodoluna* and freshwater *Aquiluna* (10%–14%) also had genes for degrading xyloglucan (alpha-D-xyloside xylohydrolase, EC 3.2.1.177) and arabinoxylan (arabinoxylan arabinofuranohydrolase, EC 3.2.1.55) that were lacking from saltwater *Aquiluna*. Xyloglucan and arabinoxylan are major components of hemicellulose in terrestrial plants [98]. The utilisation of cellulose degradation products is also supported by the prevalence of cellobiose transport system genes in freshwater members of the Luna-1 subcluster (*cebEFG*, 59% *Rhodoluna* and 43% freshwater *Aquiluna* versus 3% saltwater *Aquiluna*; Fig. 4A). In addition, a greater proportion of freshwater-adapted Luna-1 subcluster taxa harbor mechanisms for the transport of arabinogalactan or maltooligosaccharides (GanPQ; 58% *Rhodoluna* and 71% freshwater *Aquiluna* versus 22% saltwater *Aquiluna*) and fructose (FruAB; 28% *Rhodoluna* and 50% freshwater *Aquiluna* versus 4% saltwater *Aquiluna*). Thus, members of the Luna-1 subcluster from freshwater habitats have additional capabilities to utilize structural components (i.e. hemicellulose) primarily found in terrestrial habitats, as well as differing in their capacity to uptake carbon substrates.

### Widespread encoding of actinorhodopsin and heliorhodopsin across the Luna-1 subcluster is differentiated by habitat salinity

**Figure 6:**
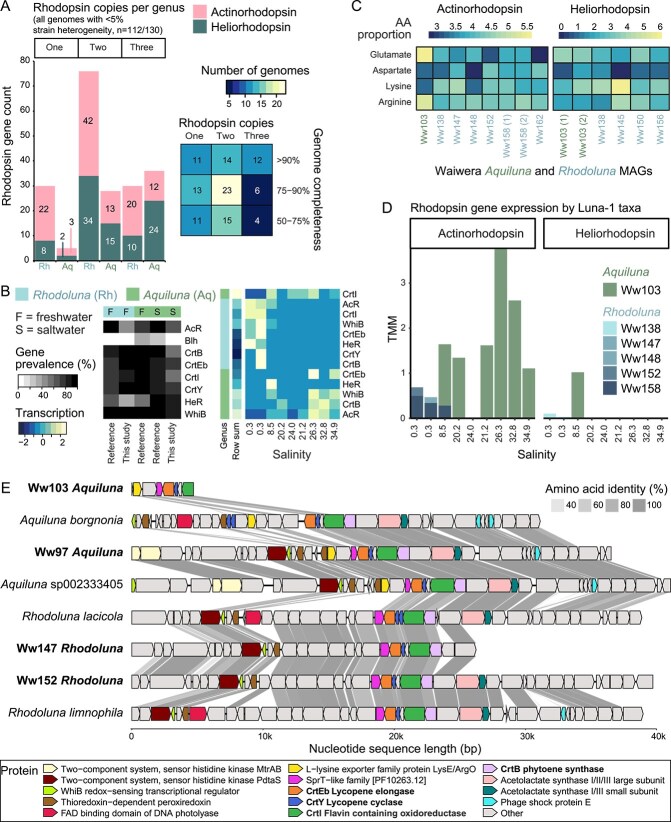
Rhodopsin and carotene synthesis gene distributions between Luna-1 subcluster genera. (A) Rhodopsin copy numbers (for all genomes with <5% strain heterogeneity). Left plot: Number of rhodopsin genes per Luna-1 subcluster genus based on GTDB representative genomes, grouped by genome encoding one, two, or three rhodopsin genes. Right plot: Relationship between genome completeness and the number of rhodopsin genes encoded by GTDB representative genomes. (B) Percentage of genomes per taxa group (*Rhodoluna*, freshwater *Aquiluna*, and saline-water *Aquiluna*) that encode proteins associated with rhodopsin and carotene synthesis (left plot), and transcription of associated genes (right plot). Transcript values (TMM) were summed by genus per gene per sampling site. Transcript values were also scaled by row with mean equal to zero and a standard deviation of one, and rows were ordered based on row means. Both plots were created using heatmap.2. (C) Charged amino acid proportions encoded by rhodopsin genes in MAGs from the Waiwera estuary. (D) Transcription of rhodopsin genes by Luna-1 subcluster members across the Waiwera estuary. (E) Synteny of carotene genes (*crtB*, *crtY*, putative *crtI*, and *crtEb*—bold font) and other associated genes in estuarine Luna-1 MAGs with rhodopsin annotations (bold font) and in the genomes of related taxa (with good assemblies). Heatmaps were plotted using pheatmap without scaling.

**Figure 7:**
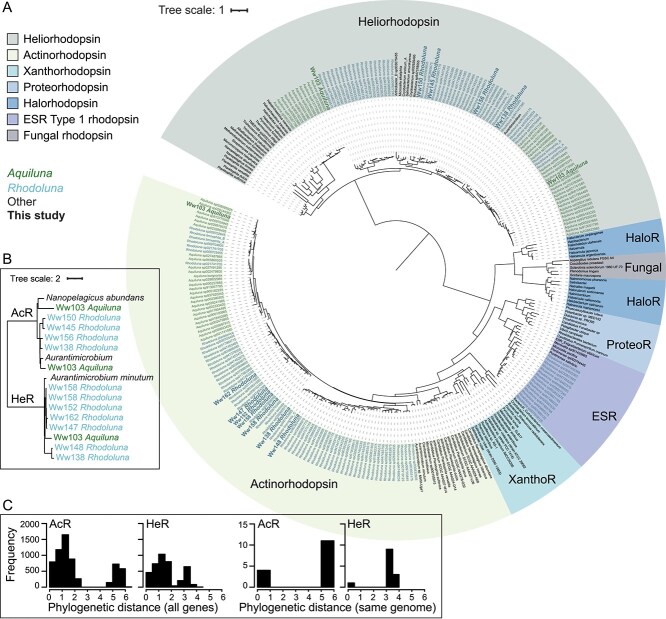
Phylogenetic placement of Luna-1 subcluster rhodopsin genes. (A) Tree based on predicted protein sequence alignments of rhodopsins from Luna-1 MAGs derived from the Waiwera estuary (larger bold font) and GTDB reference genomes, and from other taxa with known helio-, actino-, xantho-, proteo-, halo-, ESR, and fungal rhodopsins (best-fit model Q.Pfam+R7). (B) Subsetted tree showing the phylogeny of rhodopsin genes in Luna-1 subcluster MAGs from the estuary, including two evolutionarily distinct copies of HeR in *Aquiluna* MAG Ww103. (C) Pairwise phylogenetic distances between the *AcR* and *HeR* genes of Luna-1 subcluster members in the full tree. Comparisons per MAG use only MAGs with <5% strain heterogeneity.

Photoheterotrophs can use light energy harvested via bacteriochlorophyll pigments or rhodopsin proteins to supplement their organic carbon-based metabolism [99–101]. Members of the *Microbacteriaceae* lineage, which encompasses the Luna-1 subcluster, are known to host Type-1 rhodopsins unique to *Actinomycetota* bacteria, aptly named actinorhodopsin (AcR) [102, 103]. Here, we found almost all *Rhodoluna* and *Aquiluna* genomes (*n* = 118 or 91%) harbor rhodopsin genes necessary for photoheterotrophy (Supplementary Table S7), and most have two or three rhodopsin genes (e.g. 63% of 41 genomes >90% complete and with <5% strain heterogeneity, Fig. 6A). Moreover, many genomes (*n* = 74 or 57% of all genomes) encoded two distinct opsin types: heliorhodopsin (HeR) in addition to actinorhodopsin (AcR) (Supplementary Table S11). HeR is found in diverse microbial lineages, including archaea, bacteria, and algal viruses, and from varied habitats [104]. Phylogenetic analysis of genes from multiple known rhodopsin types (helio-, actino-, xantho-, proteo-, ESR, halo−/archaeal, and fungal rhodopsins) confirm the Luna-1 subcluster sequences cluster with both HeR and AcR sequences (Fig. 7A).

Although AcR and HeR genes were found in both genera, they encoded for differing ratios of rhodopsin types depending on habitat salinity. Of the Luna-1 subcluster genomes with three rhodopsin genes (18% of genomes with <5% strain heterogeneity), each *Rhodoluna* genome hosted two AcRs and one HeR copy (*n* = 10 genomes; Fig. 6A, Supplementary Table S11). In comparison, each *Aquiluna* (predominantly those from saltwater) hosted a second copy of HeR and one AcR copy (*n* = 12 genomes; 11/12 from saltwater). Saltwater *Aquiluna* overall exhibited a greater preference for heliorhodopsin (96% of genomes with at least one copy) than freshwater *Aquiluna* (57%) or *Rhodoluna* (60%) (Fig. 6B, Supplementary Table S7). Multiple genes of the same rhodopsin type per genome were potentially derived from distinct evolutionary processes. Phylogenetic analyses of these gene pairs showed a bimodal distribution in relatedness based on phylogenetic distance (PD, <1 or > 3 [HeR] and > 5 [AcR]; Fig. 7B and C). For example, estuarine MAG *Rhodoluna* Ww158 had two highly similar AcR gene copies (PD 0.1; Fig. 7B), and *Aquiluna* sp002333405 (GCA_002333405.1) had phylogenetically similar, syntenous HeR genes (PD 0.5; Fig. 7A). Both could reflect gene duplication events (although acquisition from close relatives cannot be excluded). However, the majority of second rhodopsin copies per genome were phylogenetically distinct (12/13 HeR and 12/16 AcR). Two HeR genes in the *Aquiluna* Ww103 MAG, for example, were evolutionarily distant (PD 3.4; Fig. 7B) and had unique charged amino acid profiles related to osmoprotection, as discussed above (Fig. 6C). Likewise, a number of clearly distinct bacteriorhodopsin genes (“AcR” and “ESR Type-1” rhodopsin clades; Fig. 7A) were found in several *Rhodoluna* species (e.g. *Rhodoluna* sp016462375, sp030831605, sp030832065, and sp947454255), suggesting lateral acquisition of a second copy by a common ancestor.

Genes for both rhodopsin types were transcribed by *Rhodoluna* and *Aquiluna* within the estuary (Fig. 6B and D). Despite the tendency for saltwater *Aquiluna* to harbor more HeR genes, *Aquiluna* AcR transcription was higher in the estuary, and HeR transcription was largely restricted to the freshwater-to-brackish transitional site (at 8.5 ppt; Fig. 6D). Both types were predominantly transcribed by a single population (Ww103 *Aquiluna*), and results suggest environmental modulation of rhodopsins. Microbial rhodopsins perform a variety of light-driven functions, serving as ion pumps, channels, and photosensory receptors [105–107]. AcRs are a unique outward H^+^ pump opsin (activated by green light) found only in aquatic *Actinomycetota*, particularly members of the typically freshwater acI lineage [102, 103]. HeR is the most recently described type and is evolutionarily unique, sharing <15% sequence identity with Type-1 (microbial) and Type-2 (animal) rhodopsins [104], and found in reverse orientation within the membrane [104, 108]. It has been proposed that HeRs function predominantly as signaling photoreceptors, responding to green light with long photocycles characteristic of sensory rhodopsins [104], and they have been shown to regulate diverse functions, such as glutamate synthetase and ABC transporter activity [109, 110]. Rhodopsins in Luna-1 subcluster members likely serve distinct functional roles as H^+^ pumps (AcR) and sensory transducers (HeR).

### Reliance on exogenous retinal for photoheterotrophy

Although AcR and HeR have distinct activities, both rely on the production or acquisition of retinal to function [104, 111]. Retinal is a chromophore generated via the carotenoid biosynthesis pathway. The majority of *Aquiluna* and *Rhodoluna* genomes overall (82%–83%) hosted carotenoid biosynthesis pathway genes *crtB* (15-cis-phytoene synthase; GGAP → phytoene), and *crtY* (lycopene cyclase; cyclization of lycopene to β, ε- or β, β-carotene; Fig. 6A, Supplementary Tables S6–S7). Additionally, we found a gene present in 78% of genomes (*n* = 102/130) adjacent to *crtB* and *crtY* genes that could encode CrtI desaturase (annotated as flavin-containing amine oxidoreductase; Fig. 6E). CrtI catalyzes the conversion of phytoene to lycopene, which can be condensed to beta-carotene via CrtY [112]. Despite the widespread occurrence of carotenoid biosynthesis genes, only 4% of *Rhodoluna* and 22% of *Aquiluna* genomes carried the *blh* gene required to convert beta-carotene to retinal (beta-carotene 15,15′-dioxygenase; beta-carotene + O_2_ → 2 all-trans retinal; Fig. 6A). These Blh-encoding taxa are *A. borgnonia*, 11 novel Luna-1 species (eight *Aquiluna* and three *Rhodoluna*), and *R. planktonica*. Consistent with Blh-encoding, *R. planktonica* uses its AcR as a light-induced proton pump in the absence of exogenous retinal [113]. No *blh* genes were found in estuarine Luna-1 subcluster MAGs from this study. Even though the prevalence of *blh* genes overall was low among Luna-1 subcluster members, a greater prevalence of putative retinal producers was found among members of the *Aquiluna* genus, particularly those adapted to freshwater (36% freshwater versus 15% saltwater). Likewise, when considering *Aquiluna* phylogeny (Fig. 3), and not strictly habitat-association, only members of the predominantly freshwater clade (50%), along with a few members of saline subclade A (18%), were found to encode Blh (Supplementary Table S12). This suggests phylogeny and habitat both govern the distribution of carotenoid prototrophy within the Luna-1 subcluster. However, a greater sampling of *Aquiluna* from different habitats is required to better determine the relative contributions of these factors.

The lack of *blh* in the majority Luna-1 subcluster genomes suggests most, regardless of habitat, cannot synthesize retinal *de novo*. Retinal auxotrophy has been reported for *R. lacicola* [114], and appears to be common across various taxonomic groups hosting Type-1 ion-pumping rhodopsins [114–117]. However, Type-1 rhodopsins in some of these organisms can function through the rapid binding of exogenous retinal [117, 118]. Exogenous retinal could be acquired from Blh-encoding Luna-1 prototrophs or other prototrophic taxa (e.g. actinomycetota acI clades A and B). Prototroph growth could in turn regulate auxotroph growth [103]. For example, the “boom and bust” growth cycles of auxotrophic acI clade C taxa have been attributed to exogenous retinal dependence [103]. In the Waiwera estuary, growth of Luna-1 subcluster taxa lacking *blh* genes could therefore be limited by other co-habiting taxa. Nonetheless, results show that these putative retinal auxotrophs were highly abundant and ubiquitous members of the estuary (Fig. 1A). Investigation of *blh* genes in the wider community showed numerous co-habiting bacteria could have converted beta-carotene into retinal, thereby supporting Luna-1 photoheterotrophy. A total of 132 MAGs from other lineages from the Waiwera estuary water column encoded *blh* genes, including *Actinomycetota* (*n* = 1), *Bacteroidota* (*n* = 63), and *Pseudomonadota* (*n* = 68). This included freshwater members of all three phyla (five populations, MAGs dereplicated at 98% ANI) and saltwater *Bacteroidota* and *Pseudomonadota* (30 populations), which together accounted for 8%–52% of community abundance across the estuary (Supplementary Table S12).

Instead of retinal synthesis, the carotenoid pathway in Luna-1 subcluster taxa could alternatively be used to produce flavuxanthin, an acyclic C50 carotene, via CrtEb lycopene elongase (Supplementary Tables S6–S7) [119]. The *crtEb* gene was typically located near the other three *crt* genes identified in Luna-1 genomes (Fig. 6E). Genes for phytoene synthesis (*crtB*), lycopene synthesis from phytoene (putative *crtI*), and flavuxanthin (*crtEb*) or beta-carotene (*crtY*) synthesis from lycopene were all transcribed alongside AcR by *Rhodoluna* (in freshwater) and *Aquiluna* (in saltwater) with the exception of *crtY*, for which we only observed transcription by *Rhodoluna* (Fig. 6B). Carotenoids, such as lycopene, beta-carotene, and C50 carotenoids, protect against oxidative damage and UV [120, 121]. They also give pigmentation to cells, and likely account for the red (lycopene) coloration of *Aquiluna* and *Rhodoluna* cells [27–29, 65]. A number of genes proximal to the *crt* cluster could also be involved in oxidative stress regulation, including actinobacterial *whiB* and thioredoxin-dependent peroxiredoxin; useful for organisms exposed to UV radiation [122, 123].

### Lineage–specific traits within the Luna-1 subcluster

Although a number of traits differentiate saltwater *Aquiluna* from freshwater members of the Luna-1 subcluster, others were not discriminated by habitat salinity and were instead lineage-specific, including those associated with resource uptake and utilization. When considering broad functional categories, *Rhodoluna* and *Aquiluna* MAGs from the Waiwera estuary exhibited largely similar functional potentials based on numbers of genes per KEGG pathway (Fig. 1B, Supplementary Table S13). However, *Rhodoluna* MAGs encoded significantly more genes involved in amino acid and carbohydrate metabolism, and signal transduction, with and without accounting for assembled genome length (Welch two-sample *t*-tests, *P*-values <0.05; Supplementary Table S13). A significantly greater fraction of *Aquiluna* genes with a pathway annotation were involved in translation, membrane transport, and lipid metabolism (*P*-values <0.05; Supplementary Table S13). Despite the enrichment of membrane transport genes in *Aquiluna*, *Rhodoluna* was found to harbor a greater variety in mechanisms for acquiring phosphate and iron, and comparable mechanisms for nitrogen uptake, as discussed below.

### *Rhodoluna* harbor more versatility in mechanisms for nutrient acquisition

**Figure 8:**
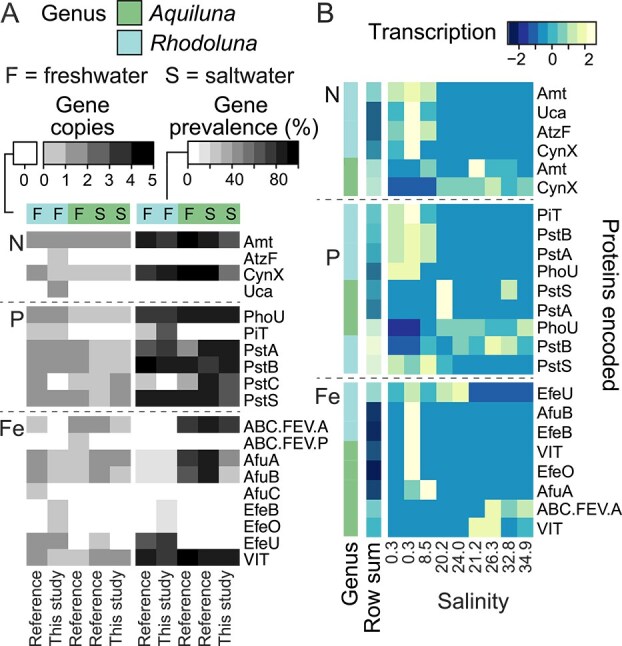
Transcription, copy numbers, and prevalence of genes for nutrient acquisition (N, P, and Fe) in Luna-1 subcluster taxa. (A) Pairs of plots showing mean gene copy numbers per genome (left) and gene prevalence per taxa group (right) for *Rhodoluna* and freshwater (F) and saltwater (S) *Aquiluna* (reference genomes and those from this study). Gene copy numbers per genome were adjusted based on genome contamination and completeness prior to calculating averages per taxa group, and are shown unscaled. Rows are ordered alphabetically based on gene name. B. Gene transcription by Luna-1 taxa in the Waiwera estuary. Transcript values (TMM-normalized counts) were summed by genus per gene per sampling site (see Supplementary Table S5 for individual gene transcription TMM values and raw counts). Transcript values were scaled by row with mean equal to zero and a standard deviation of one, and rows were ordered based on row means. Plots (A–B) were created using heatmap.2.

Pst is a high affinity phosphate transport system, and is the primary uptake system used when inorganic phosphorus is scarce (micromolar concentrations or lower) [124]. We found Pst genes (*pstABCS* and the *phoU* regulon) were present in both genera in the global Luna-1 subcluster dataset regardless of habitat (freshwater or saltwater) (Fig. 8, Supplementary Table S7). Both *Rhodoluna* and *Aquiluna* transcribed *pst* and *phoU* genes in their preferred habitats within the Waiwera estuary (Fig. 8). Phosphorus is typically a more limiting nutrient in freshwater than saltwater [2], although concentrations can be low in both habitats. Pst likely facilitates competitive uptake of low concentrations of inorganic phosphorus found across these habitats (e.g. dissolved *P* across the estuary was ≤0.6 μM in water and ≤3.3 μM in sediment porewater) [4]. Despite ubiquitous Pst encoding, there are differences between genera (and based on habitat) in how inorganic phosphorus can be acquired. In saltwater, Pst could afford *Aquiluna* with greater efficiency in phosphate uptake. Higher NaCI osmolarities are thought to stimulate ATPase-based phosphate uptake via the Pst system [125]. In contrast, about one-third of *Rhodoluna* encoded for an additional mechanism—the low-affinity Pit phosphate uptake system (35% overall: 68% of Waiwera MAGs and 27% of reference genomes). Pit-encoding genes were instead entirely absent from *Aquiluna* genomes (Fig. 8, Supplementary Table S8). Dual use of Pst and Pit can bolster phosphate uptake under low-phosphorus conditions [124], and could provide *Rhodoluna* with an advantage in freshwater. However, no *pit* gene transcription was detected in estuarine samples, suggesting the system was not used or activity was below detection. The absence of Pit-encoding genes from both freshwater and saltwater *Aquiluna* genomes suggests the adaptation is lineage-specific and that *pit* genes were acquired following divergence of the genera.

Similar lineage-specific results were evident for iron uptake mechanisms. Fe(III) predominates in oxic environments and is likely to be an important source of iron for Luna-1 subcluster members. Most freshwater and saltwater *Aquiluna* encode the FbpABC/AfuABC Fe(III) transporter system [126], where the ABC.FEV.A ATP-binding protein potentially substitutes for AfuC (75% encoded AfuA, 63% AfuB, and 80% ABC.FEV.A versus 8%–13% encoded by *Rhodoluna*; Fig. 8, Supplementary Table S8). In contrast, most *Rhodoluna* encoded the EfeU Fe(III) permease of the high-affinity EfeUOB dual Fe(II)/Fe(III) uptake system [127] (68% of *Rhodoluna* encoded EfeU versus 3% of *Aquiluna* genomes). It is unclear if EfeU operates as a dual uptake system without the canonical EfeOB binding and oxidoreductase proteins (EfeB converts Fe(II) to Fe(III)) [127] or if members of the *Rhodoluna* genus encode other proteins with these functions. If they do, the system would provide *Rhodoluna* efficient access to Fe(II) near redox gradients.

In contrast, we identified no clear difference in inorganic nitrogen uptake mechanisms based on lineage or habitat in Luna-1 subcluster taxa, despite nitrogen availability generally being higher in freshwater [2, 4]. Neither genus had annotated mechanisms for nitrate/nitrite transport (NrtA, NarK, and NasA) or assimilatory nitrate/nitrite reduction (NasBC, NarBC, NR, and NirA). Based on available annotations, inorganic nitrogen acquisition is limited to ammonium uptake (via Amt; Fig. 8), which in the Waiwera estuary could be supplied and replenished from underlying sediments where concentrations were 10-fold greater (up to 2 mM [4]) or from terrestrial runoff. In addition, most Luna-1 subcluster taxa (79% of *Rhodoluna* and 88% of *Aquiluna*) encode a putative cyanate transporter (CynX), potentially facilitating uptake of organic nitrogen. Cyanate is a form of reduced organic nitrogen that derives from various sources, including urea [128, 129]. However, no accompanying cyanase genes were annotated, which are needed to convert cyanate into ammonia [129, 130]. As such, it is unclear whether cyanate serves as a nitrogen source for either *Rhodoluna* or *Aquiluna*.

### Differences in resource acquisition and utilization between saltwater *Aquiluna* subclades

The two *Aquiluna* clades dominated by saltwater taxa (saline A and B subclusters, Fig. 3) were differentiated by several traits. Nine genes in saline A, and six in saline B, were significantly enriched (BH-adjusted *P*-value < 0.05, >25% prevalence, Supplementary Table S14). Some of these are associated with resource acquisition or utilization. For example, *Aquiluna* subclade A genomes were significantly enriched in genes encoding for the sulfur (sulfonate) NitT/TauT transport system, including substrate-binding and ATP-binding proteins (both at 35% prevalence), and the sulfonate permease SsuC, albeit at just 12% prevalence. Subclade A genomes were also significantly enriched in genes encoding a putative cation:H^+^ antiporter or Na^+^:Ca^+^ exchanger (YrbG family, 47% of genomes). Although the exact function of the encoded protein is unclear, it could potentially contribute to Na^+^ export under saline conditions if functioning as an Na^+^:H^+^ antiporter [131]. Subclade saline B was enriched in genes largely involved in cellular maintenance and processes (e.g. fatty acid and glycan biosynthesis), but also for pullulan and starch metabolism (pullulanase, 27% of genomes). Most traits differently enriched between saline subclades A and B were also enriched in freshwater *Aquiluna* (including the sulfonate transporter system and pullulanase), indicating selective retention of these traits following divergence into saltwater environments (assuming a freshwater origin for *Aquiluna*). Given the relatively small size of these subclades, further efforts to sample members could provide valuable insights into the characteristic traits and predominant habitat associations (i.e. freshwater, brackish, or marine) of these *Aquiluna* lineages.

## Conclusion

Here, we demonstrate that salinity has had a significant influence on niche differentiation in the Luna-1 subcluster, resulting in phylogenetically distinct freshwater and saltwater *Aquiluna* clades that incorporate various lineage-specific or habitat-specific traits. The most divergent traits were those associated with osmoadaptation in saltwater *Aquiluna*, where we observed an enrichment for osmoprotectant transport systems and acidic amino acid biases that point toward a salt-out osmotic stress response. A variety of other traits contributing to differences in protoheterotrophy between habitat salinities were also evident (e.g. rhodopsin preference, retinal prototrophy, and carbon uptake and utilization mechanisms). These trait differences are likely to have been driven by strong environmental selective pressures related to both salinity and resource availability in freshwater and saline habitats. Other traits appeared largely independent of habitat salinity, and diverged by lineage (e.g. phosphorus and ferrous iron acquisition strategies). Lineage-specific trait differences could enable cohabitation of genera in freshwater via distinct resource-use strategies and/or colonization of a greater range of freshwater niches. Overall, we find substantial evidence for habitat-based niche differentiation of Luna-1 subcluster genera, of which only *Aquiluna* has conclusively adapted to thrive in a brackish or marine environment.

## Supplementary Material

Supplementary_Information_ycaf122

Supplementary_Tables_ycaf122

## Data Availability

Sequence data were deposited under NCBI BioProject PRJNA668816.
