# Supplementary material for: Salinity-driven niche differentiation within the aquatic Luna-1 subcluster
Source: ISME Commun. 2025 Jul 16;5(1):ycaf122. doi: 10.1093/ismeco/ycaf122 (PMC12422013; doi:10.1093/ismeco/ycaf122)
Supplement: Supplementary_Information_ycaf122 [file supplementary_information_ycaf122.pdf]

Supplementary Information

(West *et al*., 2025, Salinity-driven niche differentiation within the aquatic Luna-1 subcluster)

Supplementary Methods

Metagenome-assembled genome (MAG) generation

Metagenomes and metatranscriptomes were generated for all samples [1]. Assembled contigs ≥2 Kbp from each site [1] were binned via MetaBAT v2.12.1 [2], MaxBin v2.2.4 [3] and CONCOCT v0.4.1 [4]. Differential contig coverages for binning were obtained using BBMap v37.93 [5] and calculated as contig length divided by total mapped read length. DAS_Tool v1.1.1 [6] was used to select the best scoring metagenome-assembled genomes (MAGs) per assembly, yielding 1,084 MAGs. CheckM v1.2.3 [7] was used to estimate strain heterogeneity and contamination (Table S1).

Multiphyla outgroup for Luna-1 phylogenetic analysis

Representative sequences of multiple phyla, other than *Actinomycetota*, were selected from the GTDB database (214 release) [8] using the criteria: type strain of species; GTDB reference; complete genome; and checkM estimates of 100% completeness, 0% contamination, and 0% strain heterogeneity (except for *Nitrospirota* at 0.91% contamination and 100% strain heterogeneity). This yielded the following representatives from 11 phyla (accession number, phylum, species):

1. GCF_000016785.1; p__Thermotogota; s__Thermotoga petrophila
2. GCF_000017405.1; p__Pseudomonadota; s__Ochrobactrum anthropi
3. GCF_003931795.1; p__Bacillota; s__Erysipelothrix piscisicarius
4. GCF_014701235.1; p__Nitrospirota; s__Dissulfurispira thermophila
5. GCF_000233775.1; p__Synergistota; s__Thermovirga lienii
6. GCF_000014145.1; p__Bacteroidota; s__Cytophaga hutchinsonii
7. GCF_000007925.1; p__Cyanobacteriota; s__Prochlorococcus marinus
8. GCF_014217355.1; p__Fusobacteriota; s__Fusobacterium hwasookii
9. GCF_000227665.2; p__Pseudomonadota; s__Thiomicrospira aerophila
10. GCF_000184345.1; p__Spirochaetota; s__Spirochaeta_A thermophila
11. GCF_000010665.1; p__Desulfobacterota; s__Solidesulfovibrio magneticus

Supplementary Figures

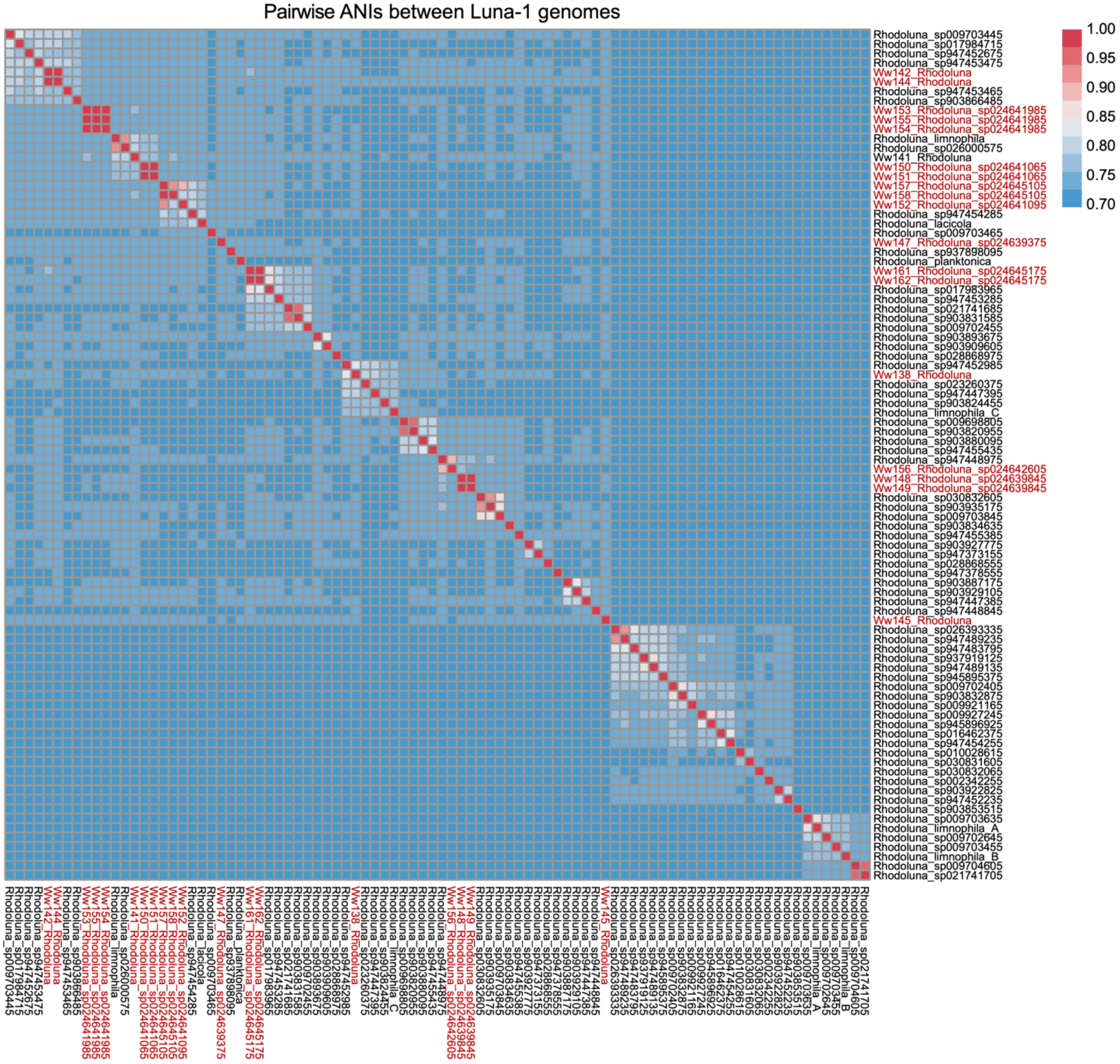

Figure S1. Pairwise average nucleotide identities (ANI) of *Rhodoluna* MAGs from the Waiwera estuary (red font) and GTDB representative genomes (black font). ANIs are based on BLAST alignments of protein-coding genes. The scale bar shows ANI as a proportion (low identity = blue, high identity = red).

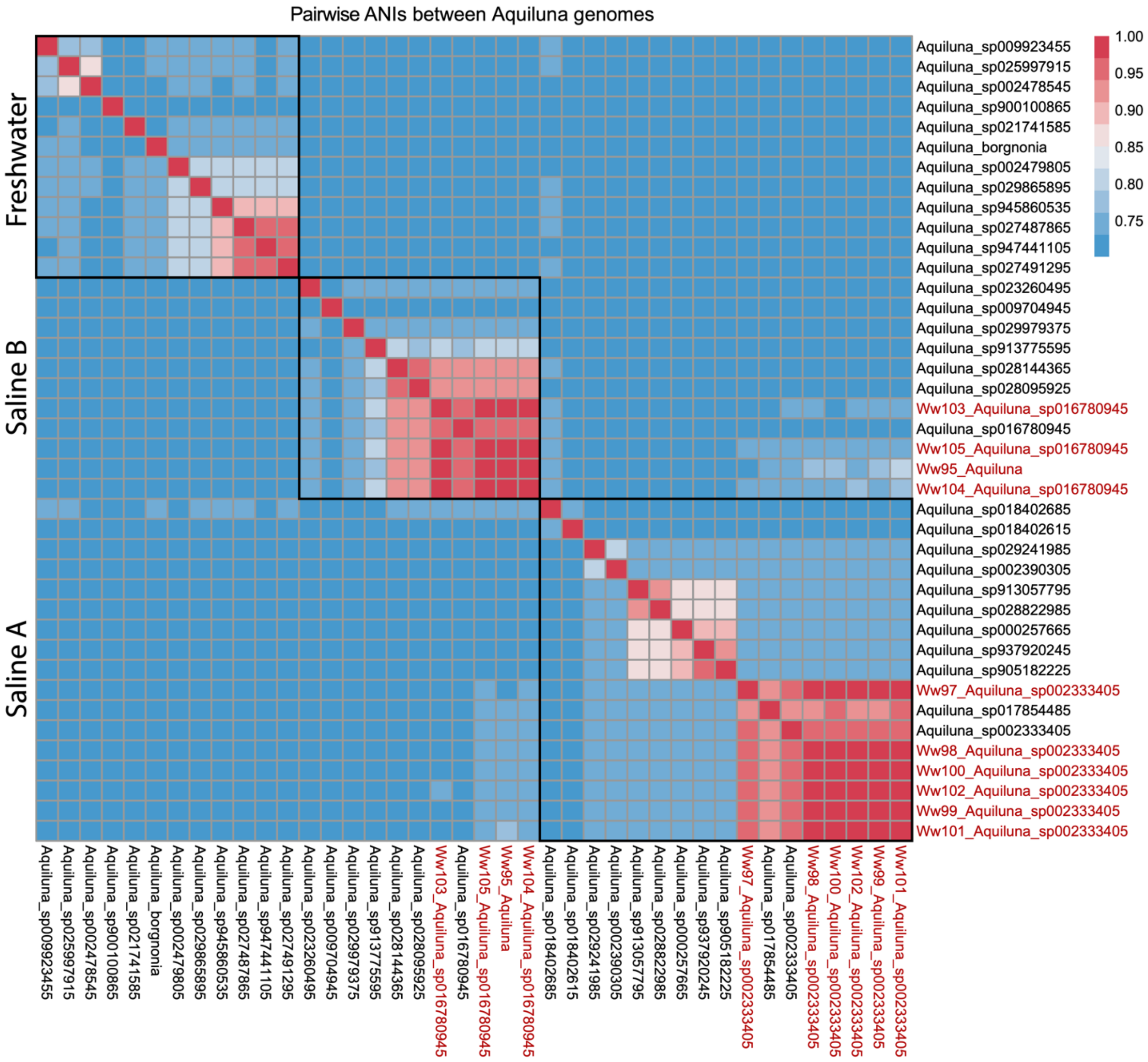

Figure S2. Pairwise average nucleotide identities (ANI) of *Aquiluna* MAGs from the Waiwera estuary (red font) and GTDB representative genomes (black font). ANIs are based on BLAST alignments of protein-coding genes. The scale bar shows ANI as a proportion (low identity = blue, high identity = red). Phylogenetic clusters dominated by freshwater and saltwater taxa (Fig. 3) are indicated by black boxes: Freshwater, Saline A and Saline B.

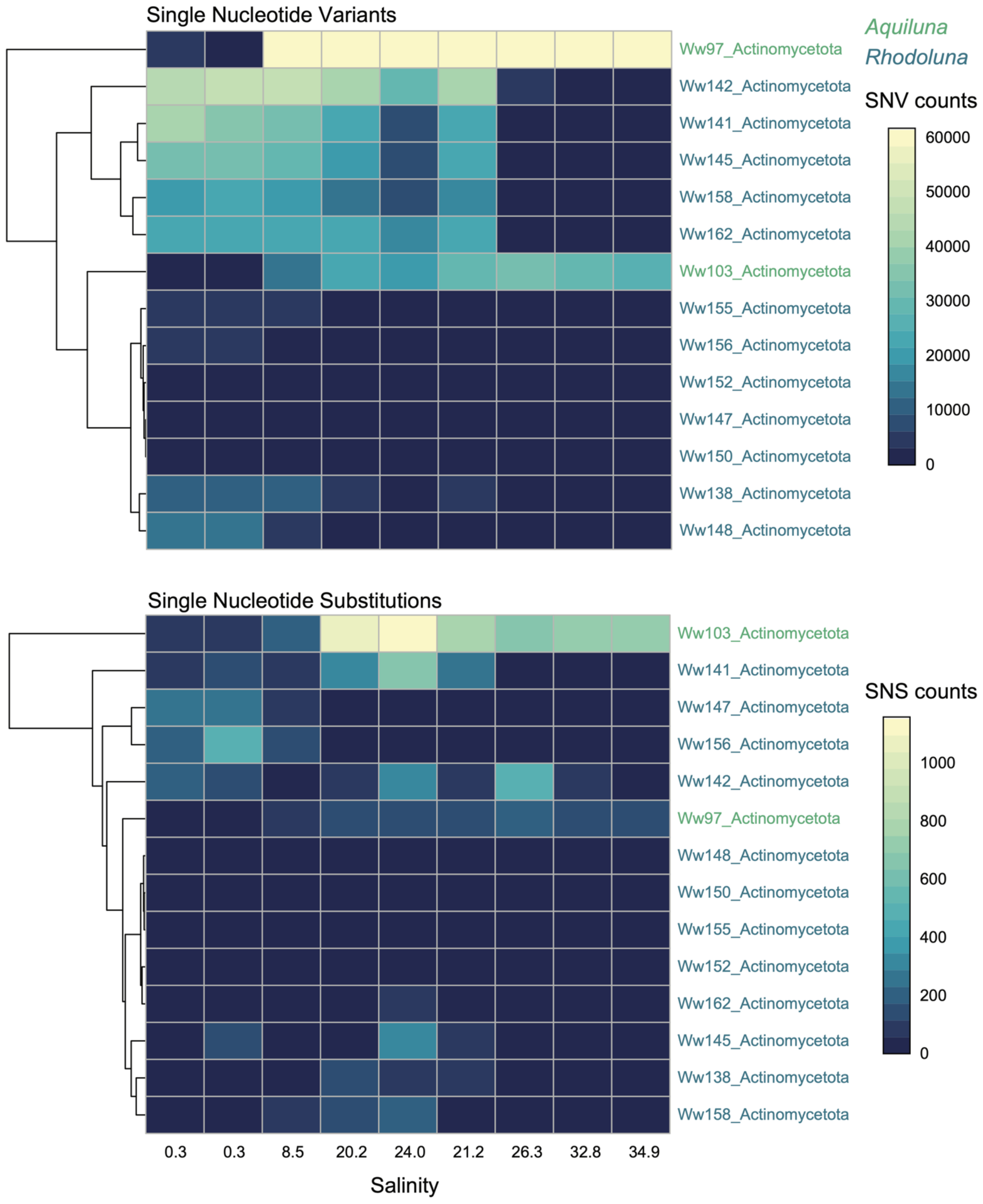

Figure S3. Number of Single Nucleotide Variants (SNV) and Single Nucleotide Substitutions (SNS) with minimum 5x coverage per dereplicated Luna-1 subcluster MAG.

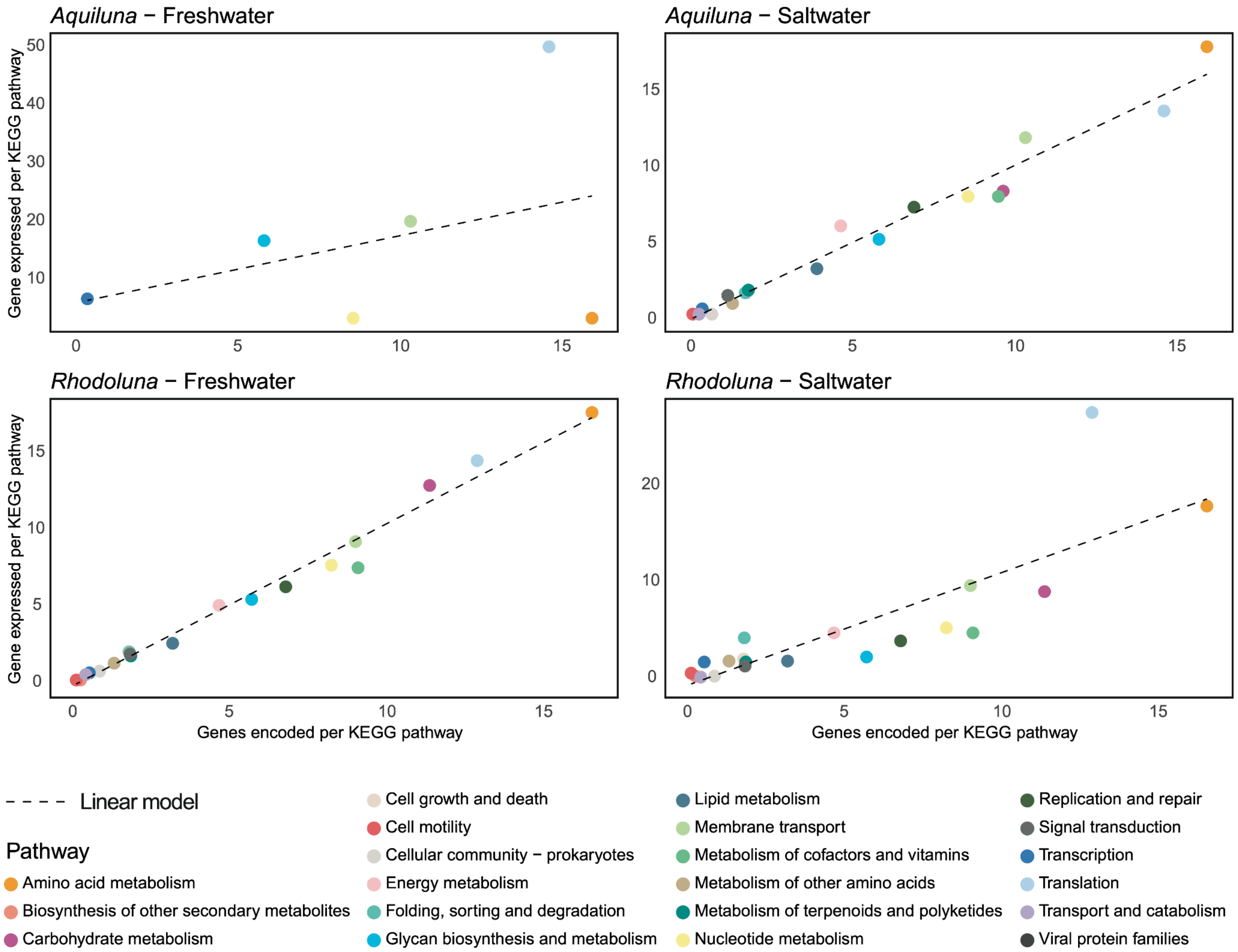

Figure S4. Relationship between the number of genes encoded per KEGG metabolic pathway versus the number of genes actively expressed per KEGG metabolic pathway for *Aquiluna* and *Rhodoluna* in freshwater and saline habitats. The dotted lines represent linear models fitted to the data with ggplot2's geom_smooth "lm" function.

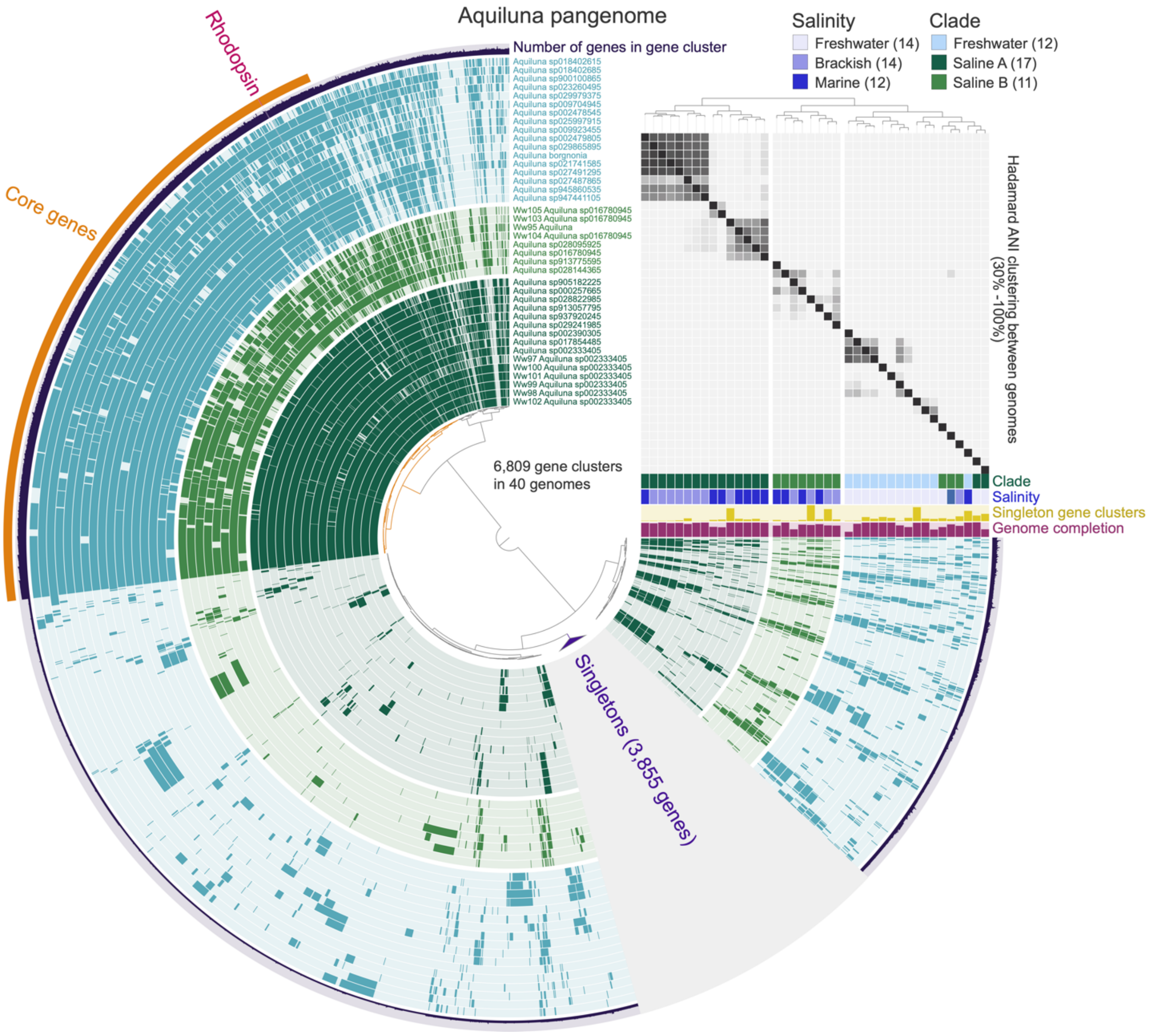

Figure S5. Pangenome analysis generated with anvi'o for *Aquiluna* MAGs and GTDB representative genomes. The layers represent individual genomes organised by Hadamard ANI clustering (high similarity = black). Clades indicated are based on the phylogenetic tree in Figure 4 and the prevailing habitat salinity of clades (Freshwater, Saline A, Saline B). Salinity of the habitats from which genomes were recovered is depicted by blue squares (darker blue = higher salinity). There was a clear demarcation of core genes that clustered together, with those in the orange cluster being highly prevalent within 70% of all *Aquiluna* genomes. The number of genes present in each gene cluster is shown in purple. Genes found only in one genome (3,855 singletons) were collapsed for better visualisation of accessory and core gene clusters.

Supplementary References

1. Tee HS, Waite D, Lear G, Handley KM. Microbial river-to-sea continuum: gradients in benthic and planktonic diversity, osmoregulation and nutrient cycling. *Microbiome*. 2021; **9:** 190.
2. Kang DD, Froula J, Egan R, Wang Z. MetaBAT, an efficient tool for accurately reconstructing single genomes from complex microbial communities. *PeerJ*. 2015; **3:** e1165.
3. Wu Y-W, Simmons BA, Singer SW. MaxBin 2.0: an automated binning algorithm to recover genomes from multiple metagenomic datasets. *Bioinformatics*. 2016; **32:** 605-607.
4. Alneberg J, Bjarnason BS, de Bruijn I, Schirmer M, Quick J, Ijaz UZ et al. Binning metagenomic contigs by coverage and composition. *Nat Methods*. 2014; **11:** 1144-1146.
5. Bushnell B. BBMap: A fast, accurate, splice-aware aligner. Lawrence Berkeley National Laboratory, Berkeley, CA, United States, 2014.
6. Sieber CMK, Probst AJ, Sharrar A, Thomas BC, Hess M, Tringe SG et al. Recovery of genomes from metagenomes via a dereplication, aggregation and scoring strategy. *Nat Microbiol*. 2018; **3:** 836.
7. Parks DH, Imelfort M, Skennerton CT, Hugenholtz P, Tyson GW. CheckM: assessing the quality of microbial genomes recovered from isolates, single cells, and metagenomes. *Genome Res*. 2015; **25:** 1043-1055.
8. Stambuk BU, da Silva MA, Panek AD, de Araujo PS. Active alpha-glucoside transport in *Saccharomyces cerevisiae*. *FEMS Microbiol Lett*. 1999; **170:** 105-110.
